# Establishment of a CoMFA Model Based on the Combined Activity of Bioconcentration, Long-Range Transport, and Highest Infrared Signal Intensity and Molecular Design of Environmentally Friendly PBB Derivatives

**DOI:** 10.3390/polym13030356

**Published:** 2021-01-22

**Authors:** Luze Yang, Minghao Li, Miao Liu

**Affiliations:** 1. College of New Energy and Environment, Jilin University, Changchun 130012, China; yanglz19@mails.jlu.edu.cn; 2 The Moe Key Laboratory of Resources and Environmental Systems Optimization, North China Electric Power University, Beijing 102206, China; limh8765@hotmail.com

**Keywords:** polybrominated biphenyls, bioconcentration, long-range transport, infrared spectrum

## Abstract

In the current study, a comparative molecular field analysis (CoMFA) model with the combined activity of polybrominated biphenyls (PBBs) bioconcentration, long-range transport, and the highest infrared signal intensity (weight ratio of 5:4:1) was constructed based on the threshold method and was further evaluated and analyzed. PBB-153 derivatives with improved combined activity values of bioconcentration, long-range transport, and the highest infrared signals intensity were designed based on contour maps of the CoMFA model. The environmental stability and functionality of the derivatives were also evaluated. The constructed model showed good prediction ability, fitting ability, stability, and external prediction ability. The contribution rates of electrostatic and steric fields to the combined activity of PBBs were 53.4% and 46.6%, respectively. Four PBB-153 derivatives with significantly improved bioconcentration, long-range transport and the highest infrared signal intensity (the combined activity value of these three parameters decreased) were screened with good environmental stability and functionality. Results validated the accuracy and reliability, and ability of the generated model to realize the simultaneous modification of the three activities of the target molecule. The binding ability of the designed derivatives to food chain biodegradation enzymes increased, thereby verifying the improvement in the bioconcentration. The half-lives of the derivatives in air and their ability to be absorbed by the plants significantly improved compared to the target molecule, further showing that the long-range transport of derivatives was reduced. In addition, the introduction of the –NO group caused the N =O stretching vibration of the derivatives to increase the infrared signal intensity. The present model provides a theoretical design method for the molecular modification of environmentally friendly PBBs.

## 1. Introduction

Polybrominated biphenyls (PBBs) are a class of brominated flame retardants with 209 homologs. Hexabromobiphenyl (PBB-153) is the main component of PBB flame retardants and is listed in Annex A of the Stockholm Convention on Persistent Organic Pollutants (POPs) [1]. As an additive flame retardant, PBBs are slowly released into the environment and taken up by the organisms [2]. Researchers have detected PBBs in mussels [3], fish [4,5,6], birds [7], etc., with the highest concentration reaching 12.4 ng/g. Even after 40 years of the Michigan pollution incident, PBBs are still detected in the human serum in the Michigan area [8]. The metabolic half-life of PBBs in the human blood is about 11 years, and the half-life in the plasma is about 12.9 to 28.7 years [9,10], indicating that they can exist in the human body for a long time. PBBs may affect thyroid function [11,12] and have estrogen-like activities [13]. Experiments on mice showed PBBs to be carcinogenic [1]; therefore, their presence in the human blood [14] and fat [15] can cause potential risks to human health. Researchers have also detected PBB-153 in lichens and orbital soils, penguins in Antarctica [16], and polar bears [17,18]. The above studies showed that PBBs have definite bioconcentration and long-range transport and are extremely harmful to both the environment and human health. Therefore, studies based on the related properties of PBBs can provide better insights into their effective mechanism and can help design better and environmentally friendly flame retardants.

Infrared spectroscopy is an important method to assess PBBs with simple functioning and no secondary pollution. The infrared spectrum is generated by the vibrational and rotational transitions of energy levels that occur when a molecule absorbs infrared radiation; that is, the molecule will absorb energy from the original ground state vibrational (rotational) kinetic energy level to the higher energy vibrational (rotational) kinetic energy level. Each molecule has its characteristic infrared spectrum, which can be analyzed qualitatively and quantitatively. The detection limit of infrared spectroscopy is limited [19]. However, PBBs are mainly present in trace amounts in nature; therefore, it is necessary to modify the molecule to improve its infrared detection sensitivity for better detection and analysis.

The 3D-QSAR model can effectively analyze and evaluate the relationship between the structure and properties of a series of compounds. Currently, the studies on 3D-QSAR are not limited to the investigation of a single activity. Previously, researchers have established 3D-QSAR models to study multiple adverse reactions of quinolones [20], two hormone activities of phthalates [21], and multiple environmental activities of polychlorinated naphthalene [22]. The multiactivity model can effectively analyze and predict multiple molecular properties at the same time. Yang et al. [23] have previously established a CoMFA model of the combined activity of infrared signal, bioconcentration, and toxicity of PBBs and successfully designed the derivatives with low bioconcentration, toxicity, and easy detection. However, this study was primarily based on the application of infrared spectroscopy to construct the model and did not focus much on the environmental aspect of the derivatives. Moreover, the proposed mechanism was ineffective in explaining the rationale behind the improved environmental activities of the target molecules. In our study, the CoMFA model of the combined activities of PBBs bioconcentration, long-range transport, and the highest infrared signal intensity was constructed based on the threshold method. PBB-153 derivatives with improved bioconcentration, long-range transport, and the highest infrared signal intensity were designed based on the information collected from the model. The environmental stability and functionality of designed derivatives were also analyzed and presented. The validation of the combined activity model and strong mechanism analysis provides a theoretical method for the molecular design of green and infrared-sensitive PBB derivatives.

## 2. Materials and Methods

### 2.1. Data Sources

The bioconcentration and long-range transport of 45 PBBs were predicted by the Toxicity Estimation Software Tool (TEST 4.2.1, released by EPA, https://www.epa.gov/chemical-research/toxicity-estimation-software-tool-test). The above properties were expressed as bioconcentration factors (BCF) and vapor pressure (VP) at 25 °C. The highest infrared signal intensity of PBBs was calculated at B3PW91/6–31G (d) level based on the density functional theory (DFT) using Gaussian 09 software [24,25].

### 2.2. Expression of Combined Activities of Bioconcentration, Long-Range Transport, and Infrared Signal Intensity of PBBs Based on the Threshold Method

The threshold method was used to characterize the combined activities of PBBs bioconcentration, long-range transport, and the highest infrared signal intensity. The formula used for the calculations is as follows:

(1) $$Y_{i}=\frac{100\times \left(X_{i}-X_{i\;\min}\right)}{\left(X_{i\;\max}-X_{i\;\min}\right)}$$

(2) $$Z=0.5\times Y_{1}+0.4\times Y_{2}-0.1\times Y_{3}$$

where X_i_ is the bioconcentration (i = 1), long-range transport (i = 2) and highest infrared signal intensity (i = 3) of the *i*th PBBs molecule, X_i min_ is the minimum value of the corresponding single activity of PBBs, X_i max_ is the maximum value of the corresponding single activity of PBBs, Y_i_ is the single activity value based on the threshold value, and Z is the combined activity value of bioconcentration, long-range transport and highest infrared signal intensity weight ratio: 5:4:1). To reduce the influence of the order of magnitude difference of single activity between different molecules, logarithm processing was performed on the single activity values of PBBs before calculation.

### 2.3. Establishment of 3D-QSAR Model of the Combined Activity of PBBs Bioconcentration, Long-Range Transport, and Infrared Signal Intensity

**Figure 1:**
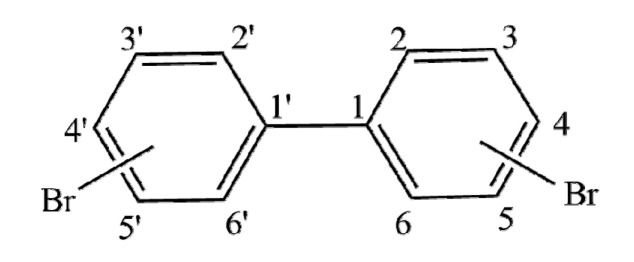
The common skeleton of polybrominated biphenyls (PBBs).

The 3D-QSAR model of the combined activity of PBBs bioconcentration, long-range transport, and the highest infrared signal intensity and the structures of 45 PBBs were constructed using Sybyl-X 2.0 software (Figure 1). The initial geometry for all molecules was generated using the Minimize module, the POWELL conjugate gradient method, and the Tripos molecular force field with energy convergence criterion set at 0.001 kcal/mol. The Gasteiger–Hückel charge was selected as the molecular charge, and the most stable conformation was obtained through 10,000 iterations. The molecule with the largest combined activity value of PBBs was used as the template molecule, and the common skeleton of all molecules was superimposed using the Align database module. Thirty-five molecules were randomly selected as the training set and the rest as the test set. Partial least squares (PLS) was used for analysis, and the one-off method was used for cross-validation of training set compounds to obtain cross-validation coefficient q^2^ and the best principal component fraction n; non-cross validation coefficient R^2^, standard deviation SEE, test value F, and the contribution rate of molecular force field were calculated using non-cross validation regression analysis. The robustness of the model was further tested and evaluated using the perturbation stability test; Q^2^, cSDEP and dq^2^/dr^2^yy were considered as the corresponding parameters. In addition, the external prediction ability of the model was tested using the cross-validation method, and SEP and r^2^_pred_ were used as evaluation parameters.

### 2.4. Molecular Stability and Functional Evaluation of PBB Derivatives Based on Gaussian Calculation

The quantum chemical parameters of target molecules and derivatives were calculated at B3PW91/6–31G (d) level based on DFT using Gaussian 09 software. The frequency [26] was used to evaluate the stability of the molecule, and the positive frequency indicated that the molecule was stable. The dissociation enthalpy of the C–Br bond represented the functional properties of PBBs as flame retardants. Theoretically, the higher the dissociation enthalpy, the better the flame retardancy.

### 2.5. Food Chain Analysis Based on Molecular Docking before and after Modification

The LibDock module of Discovery Studio 4.0 was used to dock PBBs (target molecules and their derivatives) with degradation enzymes (reductase, hydrolase, P450, and P450) in green algae, water flea, fish, and human body present in the food chain. All protein structures were obtained from the Protein Data Bank (http://www.rcsb.org). User-specified was selected for docking preferences, and max hits to save was selected as 10 to evaluate the improvement in the bioconcentration of PBBs before and after molecular modification [27].

## 3. Results and Analysis

### 3.1. Evaluation of the Combined Activity of Bioconcentration, Long-Range Transport, and Infrared Signal Intensity of PBBs

The single activity values of the bioconcentration, long-range transport, and the highest infrared signal intensity of PBBs and the combined activity values based on the threshold method are listed in Table S1. PBB-2 with the highest combined activity value was used as the template molecule to construct the 3D-QSAR model. The highest value of combined activity represented the highest bioconcentration, long-distance transport, and the lowest value of the highest infrared signal intensity.

### 3.2. Establishment and Evaluation of CoMFA Model for the Combined Activity of PBBs Molecular Bioconcentration, Long-Range Transport, and Infrared Signal Intensity

#### 3.2.1. Establishment of CoMFA Model for Combined Activity of PBBs Molecular Bioconcentration, Long-Range Transport, and Infrared Signal

The CoMFA model of the combined activity (log *Z*) of bioconcentration, long-range transport, and the highest infrared signal intensity of PBBs (hereinafter referred to as the combined activity CoMFA model) was established as dependent variables and the molecular structure of PBBs as independent variables. Model evaluation parameters of the model are shown in Table 1. The principal component of the CoMFA model was 8, and cross-validation coefficient q^2^ was 0.76 (>0.5), indicating that the established model had a good predictive ability [22]. The model standard deviation SEE was 0.06 (<0.95), F was 67.79, and non-cross validation coefficient R^2^ was 0.95 (>0.9), indicating the enhanced fitting ability of the model [28]. The scrambling stability test parameter Q^2^ was 0.53, cSDEP was 0.18, and dq^2^/dr^2^yy was 1.17 (<1.2), reflecting the good stability of the constructed model [29]. SEP and r^2^_pred_ values obtained through the external verification of the test set were 0.19 and 0.65 (>0.6), respectively, indicating that the model had a good external prediction ability [23]. The contribution rates of the electrostatic and steric fields to the combined activity of PBBs were 53.4% and 46.6%, respectively.

#### 3.2.2. Analysis of Contour Maps of Combined Activity CoMFA Model

**Figure 2:**
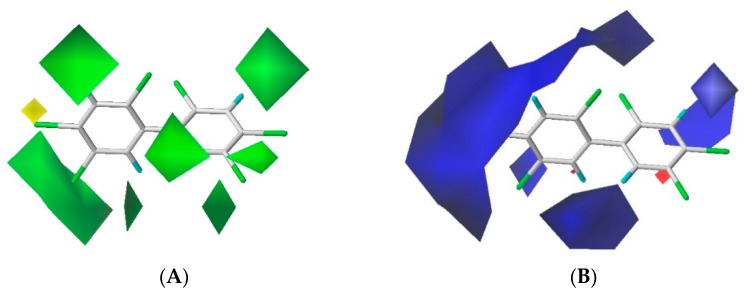
The contour maps of steric (**A**) and electrostatic field (**B**) of the combined activity comparative molecular field analysis (CoMFA) model.

The contour maps of the steric and electrostatic field of the combined activity CoMFA model for the PBB-153 target molecule are shown in Figure 2A,B, respectively. A more detailed explanation of contour maps is in the Supplementary Information. In the steric field, the green area was distributed at sites 3, 3′, 5, 5′, 6, and 6′ of the molecule, and the yellow area was mainly distributed at site 4. In the electrostatic field, the molecules were mainly covered by the blue area, and they were distributed at sites 2, 2′, 3, 3′, 4, 5, and 6. The introduction of small-volume groups into the green area of the steric field and low-electrostatic groups into the blue area of the electrostatic field reduced the combined activity of the target molecule [30]. Because the contribution rate of the electrostatic field was high, the molecular design of PBB-153 derivatives was constructed using the electrostatic field contour map preferentially.

### 3.3. Molecular Design of PBB-153 Derivatives and Model Validation

#### 3.3.1. Molecular Modification of PBB-153 Based on Combined Activity CoMFA Model

Four PBB-153 derivatives were designed and screened according to the information obtained from the contour map of the combined activity CoMFA model of PBBs (Table 2). The decreased ratio of the combined activity of derivatives was in the range of 8.02% to 27.39%. Among all the derivatives, 5-NO-5′-OCN-PBB-153 derivative molecules were most affected. 5-NO-5′-ONO-PBB-153 and 5-NO-5′-OCN-PBB-153 derivatives were both substituted by the groups with lower electrostatic properties than Br atoms at sites 5 and 5′. Moreover, as the volume of the –OCN group was smaller than –ONO, the latter had a higher combined activity value. The improved bioconcentration, long-range transport, and the highest infrared signal intensity of all designed derivatives were consistent with the information collected from the contour maps of the constructed CoMFA model.

#### 3.3.2. Validation of Combined Activity CoMFA Model

##### Validation of Combined Activity CoMFA Model through Single Activity Evaluation

In this study, a CoMFA model of the combined activity of PBBs bioconcentration, long-range transport, and the highest infrared signal intensity was constructed, and four PBB-153 derivative molecules with significantly reduced combined activity values were successfully designed based on the information of the contour maps of the model. To further verify the accuracy and reliability of the model, three single activities of PBBs bioconcentration, long-range transport, and the highest infrared signal intensity were evaluated, and the single activity CoMFA models were constructed, and the contour maps were compared. Gaussian 09 software was used to calculate the highest infrared signals intensity of PBB-153 derivatives, and the constructed bioconcentration and long-range transport single activity CoMFA models were used to predict the bioconcentration and long-range transport activities of derivatives. The resulting values of molecular bioconcentration, long-range transport, and the highest infrared signal intensity of PBB-153 derivatives are shown in Table 3. Compared to the target molecule, the designed derivatives had lower bioconcentration values, and the ratio was in the range of 3.92 to 21.63%. Moreover, 5-NO-5′-ONO-PBB-153 and 5-NO-5′-OCN-PBB-153 had relatively lower bioconcentration, which was consistent with the results of the combined activity. The long-range transport properties of the derivatives were lower than those of the target molecules, and the change rates ranged from 12.15 to 28.9%. The highest infrared signal intensity of the PBB-153 derivatives increased with the change rate from 1.96 to 7.41%. The modifications observed in the three single activities of derivatives were consistent with the combined activity model. In addition, the ratio of bioconcentration, long-range transport, and the highest infrared signal intensity of 5-NO-5′-ONO-PBB-153 derivative was around 7:6:1 and was in complete agreement with the weight ratio (5:4:1) of the constructed model, thereby validating the accuracy of the combined activity CoMFA model.

##### Validation of Combined Activity CoMFA Model Based on the Contour Maps of Single Activity and Combined Activity CoMFA Models

CoMFA models of PBBs bioconcentration, long-range transport, and the highest infrared signal intensity were established, and the contour maps of single activity and combined activity models were compared (Table 4). The contribution rates of the steric field for the three single activities were 43.7%, 43.7%, and 34.5%, respectively, and the contribution rates of the electrostatic field were 56.3%, 56.3%, and 65.5%, respectively. The electrostatic field showed high and consistent contribution rates in both the single activity model and the combined activity model.

The contour maps of the steric field of three single activities were mainly covered by the green area with varied distribution for all three activities. For instance, the green area was mainly distributed at sites 3, 3′ in the case of the bioconcentration contour map, whereas it enclosed all sites of the molecule in the long-distance contour map and was distributed near sites 2 and 3 in the infrared variation contour map. The green area in the combined activity model contour map was dispensed at sites 3, 3′, 5, 5′, 6, and 6′. The steric contour maps of the combined activity CoMFA model carried the information of all three single activities.

The three single activity electrostatic field contour maps were mainly covered by the blue area with its distribution near sites 3, 4′, 6, and 6′ in the bioconcentration contour map, at sites 2, 3, 4, 4′, 5, 5′, 6, and 6′ in the long-range transport contour map, and near sites 2, 2′, 4, 4′ and 5 in the infrared signal contour map. In the contour map of the combined activity model, the blue area was distributed at sites 2, 2′, 3, 3′, 4, 5, and 6, primarily covering the information of the three single activity contour maps. In summary, the combined activity model of PBBs bioconcentration, long-range transport, and the highest infrared signal intensity covered the information of the three single activity models, which can be modified by the combined activity of PBB-153 with certain reliability.

### 3.4. Molecular Properties Evaluation of PBB-153 Derivatives

#### 3.4.1. Environmental Stability and Functionality Evaluation of PBB-153 Derivatives

The environmental stability and functionality evaluation parameters of PBB-153 derivatives are listed in Table 5. All frequencies of the derivatives were greater than zero, indicating the existence of the environmental stability of the theoretical derivatives [31]. The C–Br bond dissociation enthalpy of derivatives was in the range of −1.76 to −0.02% compared with the target molecule and did not change in general, indicating that the derivatives retained good flame retardant properties of the target molecule and had better functional properties. Thus, it can be concluded that the designed PBB-153 derivatives had good environmental stability and functionality.

#### 3.4.2. Evaluation of Environmental Friendliness of PBB-153 Derivatives

The bioconcentration, long-range transport, toxicity, and the highest infrared signal intensity of PBB-153 were comprehensively analyzed to evaluate the environmental friendliness of the designed derivatives. Table 6 lists the logarithmic order of single activity prediction values of bioconcentration, long-range transport, and the highest infrared signal intensity. The improvement ratios of the original values of the three activities were in the range of 18.14 to 66.88%, 13.67 to 62.33%, and 88.32 to 99.39%, respectively, indicating considerably improved bioconcentration, long-range transport, and the highest infrared signal intensity of PBB-153 derivatives. Considering the toxicity in fish as an example, the biological toxicity of PBB-153 derivatives was evaluated by EPIWEB 4.1 and is shown in Table 6. LC_50_ is the median lethal concentration that governs the toxicity of a material. A higher value of LC_50_ indicates that the compound is less toxic. The PBB-153 derivatives showed a significant increase in their LC_50_ values and consequently a considerable reduction in the toxicity in fish. On the whole, the designed derivatives exhibited lower bioconcentration, long-range transport, biological toxicity, and higher infrared detection sensitivity, thus theoretically being more environmentally friendly.

### 3.5. Mechanism Analysis for Improvement of PBB-153 Derivatives in the Bioconcentration

Persistent organic pollutants (POPs) can be enriched along the food chain [32]. A natural food chain (green algae → daphnia → fish → human body) was selected to analyze the changes in bioconcentration before and after PBB-153 molecular modification, and the risk of biomagnification was evaluated. The total scores of PBB-153 and derivatives docked with four types of degrading enzymes in organisms are shown in Table 7. It was found that the total scores of all the derivatives were higher than the target molecule, suggesting that the derivatives were more easily degraded by the four organisms selected from the food chain and thus showed decreased bioaccumulation. The rising ratios of the total scores of the derivatives docked with the degrading enzymes in green algae, daphnia, fish, and the human body were 13.22–29.98%, 14.04–30.71%, 5.58–41.19% and 15.98–28.5%, respectively. Among them, 5-NO-5′-OCN-PBB-153, 5-NO-5′-ONO-PBB-153, 2-OCHO-5-NO-PBB-153, and 5-NO-5′-OCN-PBB-153 derivatives had the highest total scores. In general, green algae and daphnia have higher degradability against the derivatives. Hence, theoretically, the bioconcentration of the derivatives in these two low-level organisms in the food chain was reduced, consequently lowering the risk of transmission and further enrichment in the food chain. It is worth mentioning here that the most significant degradation derivatives in the four organisms were mainly 5-NO-5′-ONO-PBB-153 and 5-NO-5′-OCN-PBB-153. The total score for docking 5-NO-5′-OCN-PBB-153 with fish degrading enzyme was increased by 39.45% and was comparable to 2-OCHO-5-NO-PBB-153 (41.19%). The docking results were consistent with the single activity prediction results of bioconcentration, indicating lower bioconcentration values of 5-NO-5′-ONO-PBB-153 and 5-NO-5′-OCN-PBB-153. The increase in the bioaccumulation of the derivative in the organism along the food chain and the organism’s higher degradability was indicated by the increase in the total scores with degrading enzyme, as observed in the case of 5-NO-5′-ONO-PBB-153. In addition, the increase in the overall total scores of the derivatives compared to the target molecule in the food chain delivery indicated that the derivatives were more likely to be degraded during the food chain delivery process. Finally, the experiments based on the docking of PBB-153 derivatives with biodegradable enzymes in the food chain further verified the reasons behind the reduction in the molecular bioconcentration of the designed derivatives and demonstrated the potential of the molecular modification.

### 3.6. Mechanism Analysis for Improvement of PBB-153 Derivatives in Long-Range Transport

Lignin peroxidase (LiP enzyme) produced by white-rot fungi attached to the plants can oxidize and reduce POPs [33], which is a kind of phytoremediation—an economic, efficient, energy-saving, and environmentally friendly process [34]. In the present study, the long-range transport of PBB-153 before and after molecular modification was evaluated from three aspects. First, the total scores of molecules and LiP enzyme indicate the degree of absorption by plants, i.e., the possibility of entering the atmosphere. Second, the half-life in the air indicates the ability of the molecules to get oxidized in the atmosphere hence, their stability in the atmosphere. Lastly, the vapor pressure of the molecules indicates their long-range transport capacity. The higher the vapor pressure, the higher is the volatility and easier the long-distance migration of the molecule.

The total scores of PBB-153 derivatives docking with the LiP enzyme were higher than those of the target molecule and ranged from 3.9 to 41.15%. The changing trend was consistent with the trend predicted by the vapor pressure single activity. This suggested that the designed derivatives were more likely to be oxidized by bacteria present in plants, thereby reducing their possibility of entering the air. The AopWin v1.92 method of the EPIWEB 4.1 database was used to evaluate the ability of PBB-153 and its derivatives to get oxidized in the atmosphere and to further determine the long-range transport after modification (Table 8). The half-life of the target molecule in the air was about 83 days, and that of the designed derivatives varied from 4.01 to 5.75 days, and the reduction ratio of the target molecule ranged from 93.07 to 95.17%. Among the designed derivatives, the 5,5′-NO-PBB-153 had the longest half-life, whereas single-activity evaluation showed that it had a lower vapor pressure. 5-NO-5′-ONO-PBB-153 and 5-NO-5′-OCN-PBB-153 also showed lower vapor pressure and significantly reduced half-lives. This confirmed that modified PBB-153 molecules had lower long-range transport, which was also consistent with the prediction results of the combined activity CoMFA model. Altogether, the designed derivatives were readily degraded by the plants, oxidized in the atmosphere, and had lower volatilization ability and thus reduced long-range transport.

### 3.7. Mechanism Analysis for Improvement of PBB-153 Derivatives in the Highest Infrared Signal Intensity

**Figure 3:**
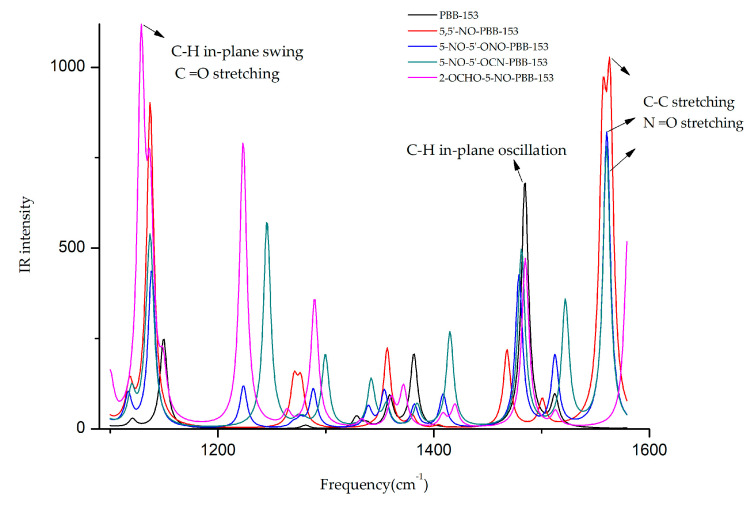
Infrared spectra of different frequencies (1100–1600 cm^−1^) before and after modification of PBB-153.

The infrared spectra of the target molecule and four derivatives at different frequencies are shown in Figure 3. It was observed that the frequencies corresponding to the highest infrared signal of all derivatives were changed. The highest infrared signal intensities of 5,5′-NO-PBB-153 and 2-OCHO-5-NO-PBB-153 increased significantly, and the corresponding signal frequencies of 5,5′-NO-PBB-153, 5-NO-5′-ONO-PBB-153, and 5-NO-5′-OCN-PBB-153 derivatives were comparable. The frequency and signal type of the target molecule and its derivatives were analyzed by GaussView 5.0 software at the highest value of the infrared signal intensity [35]. The frequency of the highest infrared signal in the PBB-153 molecule was 1484.53 cm^−1^ and was mainly due to the in-plane oscillation of C–H on the two benzene rings. When the infrared signal of the 5,5′-NO-PBB-153 derivative molecule was the strongest, the value of its frequency was 1563.32 cm^−1^, primarily caused by the C–C stretching vibration on the two benzene rings and the N =O stretching vibration on the –NO group. The frequencies of 5-NO-5′-ONO-PBB-153 and 5-NO-5′-OCN-PBB-153 at the strongest infrared signal were 1560.34 cm^−1^ and 1560.21 cm^−1^, respectively, mainly caused by the unilateral C–C stretching vibration on the benzene ring and the N=O stretching vibration on the –NO group. The frequency of the highest infrared signal of 2-OCHO-5-NO-PBB-153 was 1128.83 cm^−1^, which mainly resulted from the in-plane swing vibration of C–H on both sides of the benzene ring and the C=O stretching on the –OCHO group.

The –NO groups were introduced in all the designed derivatives. The vibration type at the highest signal intensity before and after the modification of the PBB-153 molecule was changed from C–H in-plane rocking vibration to infrared vibration of newly introduced groups. The introduction of –NO groups caused N=O stretching vibrations in the derivative molecules (5,5′-NO-PBB-153, 5-NO-5′-ONO-PBB-153, and 5-NO-5′-OCN-PBB- 153) with increased infrared signal intensity and frequency in the range of 1560.21 to 1563.32 cm^−1^. Moreover, the introduction of the –OCHO group had a higher impact on the highest infrared signal intensity of the molecule than the –NO group; hence, both –OCHO and –NO group substituted 2-OCHO-5-NO-PBB-153 derivative showed the highest infrared signal intensity as a result of C=O stretching vibrations.

## 4. Conclusions

In summary, a CoMFA model of the combined activity of PBBs bioconcentration, long-range transport, and the highest infrared signal intensity was constructed. Based on this model, four environmentally friendly PBB-153 derivatives were designed. The bioconcentration, long-range transport, and the highest infrared signal intensity of derivatives significantly improved. The refined values were further verified by studying their food chain enrichment, half-life calculation, molecular docking, and Gauss calculation. In addition, we provided theoretical support for the study of pollution control of POPs.

## Figures and Tables

**Table 1:** Evaluation parameters of the combined activity model.

Model	q^2^	n	SEE	R^2^	F	r^2^_pred_	SEP	Q^2^	cSDEP	dq^2^/dr^2^yy
CoMFA	0.76	8	0.06	0.95	67.79	0.65	0.19	0.53	0.18	1.17

**Table 2:** The combined activity of bioconcentration, long-range transport, and the highest infrared signal before and after the modification of hexabromobiphenyl (PBB-153).

No.	Molecule	Combined Activity Value	Change Rate (%)
0	PBB-153	1.11	
1	5,5′-NO-PBB-153	1.02	8.29
2	5-NO-5′-ONO-PBB-153	0.87	21.80
3	5-NO-5′-OCN-PBB-153	0.81	27.39
4	2-OCHO-5-NO-PBB-153	1.02	8.02

**Table 3:** The single activity value and change rate of PBB-153 derivatives bioconcentration, long-range transport, and highest infrared signal intensity.

**No.**	Molecule	Log (IR Intensity)	Change Rate	Log (BCF)	Change Rate	Log (VP)	Change Rate (%)
0	PBB-153	2.84		2.22		−7.67	
1	5,5′-NO-PBB-153	3.01	5.98	1.87	−15.59	−9.89	−28.90
2	5-NO-5′-ONO-PBB-153	2.92	2.79	1.78	−19.87	−9.09	−18.49
3	5-NO-5′-OCN-PBB-153	2.89	1.96	1.74	−21.63	−9.26	−20.74
4	2-OCHO-5-NO-PBB-153	3.05	7.41	2.13	−3.92	−8.60	−12.15

**Table 4:** The single activity and combined activity contour maps of PBBs bioconcentration, long-range transport, and the highest infrared signal intensity.

CoMFA Model	Combined Activity	Bioconcentration Activity	Long-Range Transport Activity	Infrared Intensity Activity
Steric field	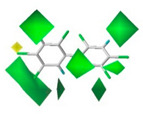 46.6%	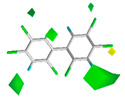 43.7%	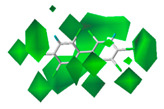 43.7%	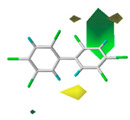 34.5%
Electrostatic field	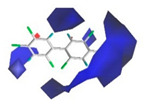 53.4%	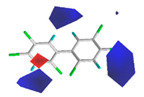 56.3%	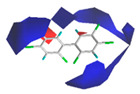 56.3%	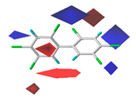 65.5%

**Table 5:** Calculation of frequency and C–Br bond dissociation enthalpy before and after PBB-153 modification.

No.	Molecule	Frequency	C–Br Bond Dissociation Enthalpy	Change Rate (%)
0	PBB-153	20.27	83.37	
1	5,5′-NO-PBB-153	22.68	82.04	−1.59
2	5-NO-5′-ONO-PBB-153	22.84	82.94	−0.52
3	5-NO-5′-OCN-PBB-153	21.88	81.90	−1.76
4	2-OCHO-5-NO-PBB-153	16.80	83.35	−0.02

**Table 6:** Prediction of molecular toxicity before and after PBB-153 modification.

No.	Molecule	LC_50_	Change Rate (%)
0	PBB-153	0.00022	
1	5,5′-NO-PBB-153	0.01	5404
2	5-NO-5′-ONO-PBB-153	0.05	22,836
3	5-NO-5′-OCN-PBB-153	0.17	78,799
4	2-OCHO-5-NO-PBB-153	0.24	111,827

**Table 7:** Total scores of PBB-153 molecular docking with food chain degrading enzyme before and after modification.

**No.**	Molecule	Total Scores (Green Algae)	Change Rate (%)	Total Scores (Daphnia)	Change Rate (%)	Total Scores (Fish)	Change Rate (%)	Total Scores (Human Body)	Change Rate (%)
0	PBB-153	49.88		45.80		47.17		80.19	
1	5,5′-NO-PBB-153	62.52	25.34	64.89	41.68	49.80	5.58	93.01	15.98
2	5-NO-5′-ONO-PBB-153	56.47	13.22	59.86	30.71	58.88	24.81	102.00	27.19
3	5-NO-5′-OCN-PBB-153	64.83	29.98	52.23	14.04	65.78	39.45	103.05	28.50
4	2-OCHO-5-NO-PBB-153	60.11	20.52	52.45	14.53	66.60	41.19	96.54	20.38

**Table 8:** Calculation of half-life in air and total scores of PBB-153 docking with lignin peroxidase (LiP) enzyme before and after modification.

No.	Molecule	T_1/2_ (day)	Change Rate (%)	Total Scores (LiP Enzyme)	Change Rate (%)
0	PBB-153	82.96		57.70	
1	5,5′-NO-PBB-153	5.75	93.07	81.45	41.15
2	5-NO-5′-ONO-PBB-153	4.01	95.17	78.94	36.80
3	5-NO-5′-OCN-PBB-153	4.21	94.92	81.23	40.77
4	2-OCHO-5-NO-PBB-153	4.86	94.14	59.95	3.90

## Data Availability

Data is contained within the article or supplementary material.

## Supplementary Materials

The following are available online at https://www.mdpi.com/2073-4360/13/3/356/s1, Table S1: The single activity values of the bioconcentration, long-range transport and the highest infrared signal intensity and the combined activity values of PBBs.
