# Supplementary material for: Establishment of a CoMFA Model Based on the Combined Activity of Bioconcentration, Long-Range Transport, and Highest Infrared Signal Intensity and Molecular Design of Environmentally Friendly PBB Derivatives
Source: Polymers (Basel). 2021 Jan 22;13(3):356. doi: 10.3390/polym13030356 (PMC7865581; doi:10.3390/polym13030356)
Supplement: Supplementary file 1 [file polymers-13-00356-s001.pdf]

polymers

MDPI

Supplementary information

(Manuscript ID: polymers-1062236)

Establishment of a CoMFA Model Based on the Combined Activity of Bioconcentration, Long-range Transport, and Highest Infrared Signal Intensity and Molecular Design of Environmentally Friendly PBB Derivatives

Luze Yang [1], Minghao Li [2] and Miao Liu [1,*]

1. College of New Energy and Environment, Jilin University, Changchun 130012, China; yanglz19@mails.jlu.edu.cn
2. The Moe Key Laboratory of Resources and Environmental Systems Optimization, North China Electric Power University, Beijing 102206, China; Email: limh8765@hotmail.com

* Correspondence: liumiao@jlu.edu.cn

Table S1. The single activity values of the bioconcentration, long-range transport and the highest infrared signal intensity and the combined activity values of PBBs.

No.	Molecule	Frequency (cm^{-1})	Highest infrared signal intensity (km/mol)	BCF	VP (25°C)	Combined activity value (Z)	logZ
1	PBB-1	767.75	228.21	731.08	0.00268	81.32	1.91
2	PBB-2	772.50	216.09	943.58	0.00302	88.00	1.94
3*	PBB-3	777.04	176.12	643.31	0.0028	79.68	1.90
4	PBB-7	1509.71	343.00	849.64	4.1E-05	73.11	1.86
5	PBB-11	798.57	215.16	624.42	0.00013	70.75	1.85
6	PBB-18	1502.33	267.83	267.23	6.1E-06	42.23	1.63
7	PBB-21	1475.23	327.94	768.46	6.5E-06	66.66	1.82
8	PBB-26	1029.10	283.70	710.55	0.00047	75.47	1.88
9	PBB-30	749.47	231.10	478.68	9.4E-06	57.96	1.76
10*	PBB-31	1034.41	259.71	286.25	5.9E-06	43.92	1.64
11*	PBB-39	756.22	239.75	383.85	9.8E-06	52.57	1.72
12	PBB-41	776.66	146.28	544.15	1.7E-06	59.36	1.77
13	PBB-45	760.52	188.34	379.28	1.2E-06	48.66	1.69
14	PBB-48	1492.73	409.22	762.55	1.7E-06	62.21	1.79
15	PBB-51	737.14	178.51	706.13	6.1E-07	62.24	1.79
16*	PBB-54	741.10	342.90	370.95	3.1E-07	41.89	1.62
17	PBB-59	1471.81	290.82	219.60	2.2E-06	34.70	1.54
18	PBB-61	1436.77	355.22	602.65	5.6E-07	54.72	1.74
19	PBB-67	1491.96	550.64	261.11	1.7E-06	34.98	1.54
20*	PBB-78	756.95	247.05	352.80	9.4E-07	44.95	1.65
21	PBB-81	758.39	207.14	227.24	9E-07	35.20	1.55
22	PBB-88	1362.18	299.68	225.16	1.6E-07	29.03	1.46
23	PBB-91	818.12	201.33	228.75	2E-07	31.94	1.50
24	PBB-95	1021.84	323.14	300.16	1.5E-07	35.35	1.55
25*	PBB-101	1491.25	507.82	448.20	6.9E-08	40.91	1.61
26*	PBB-105	1473.34	509.67	117.37	9.6E-08	9.53	0.98
27*	PBB-111	1598.92	340.97	237.04	1.8E-07	29.94	1.48
28*	PBB-118	1489.83	689.41	189.96	1.1E-07	19.93	1.30

29	PBB-121	746.64	288.42	176.26	2.4E-07	24.31	1.39
30	PBB-126	1463.10	420.64	243.98	1.9E-07	29.66	1.47
31	PBB-132	1463.23	393.54	190.26	2.2E-08	19.05	1.28
32	PBB-142	1349.29	510.16	429.40	1.3E-08	35.93	1.56
33	PBB-152	1425.34	422.49	295.93	7.7E-08	32.16	1.51
34	PBB-153	1484.53	688.69	165.55	2.1E-08	12.74	1.11
35	PBB-169	1584.99	368.14	186.46	1.1E-08	17.17	1.23
36	PBB-173	757.17	521.77	343.02	7.3E-09	29.14	1.46
37	PBB-178	1350.60	346.71	318.18	6.1E-09	29.01	1.46
38	PBB-180	1028.93	444.28	234.07	4.5E-09	19.66	1.29
39	PBB-182	1438.97	472.30	311.78	4.6E-09	26.26	1.42
40	PBB-193	1430.63	406.71	318.24	6.3E-09	28.29	1.45
41	PBB-194	1422.35	637.17	264.14	5.9E-10	15.99	1.20
42	PBB-200	1425.27	520.98	311.57	6.2E-10	21.09	1.32
43*	PBB-205	1392.36	577.33	236.38	6.3E-10	14.01	1.15
44	PBB-208	1351.58	854.05	330.51	1.8E-10	17.16	1.23
45	PBB-209	1348.99	1031.20	384.81	1E-10	18.48	1.27

* represents the test set of the CoMSIA model, the remainder is the training set.

3.2.2. Analysis of Contour Maps of Combined Activity CoMFA Model

Supplementary information of contour maps: The CoMFA model consists of two types of contour maps: steric and electrostatic field. The sterically favorable regions are represented in green and the unfavorable regions in yellow in the contour map of steric field, which means the introduction of large and small volume groups into green and yellow area will increase the effect value, respectively. The electrostatic field is denoted by blue and red contours, where the blue regions represent the electropositive groups near these regions favorable to effect and the red regions indicate that the electronegative groups close to these regions may increase effect value [1].

References

[1] Xu, Z.; Chen, Y.; Qiu, Y.L.; Gu, W.W.; Li, Y. Prediction of stability for polychlorinated biphenyls in transformer insulation oil through three-dimensional quantitative structure-activity relationship pharmacophore model and full factor experimental design. *Chem. Res. Chin. Univ.* **2016**, *32*, 348-356.
